# Physiological and molecular bases of the nickel toxicity responses in tomato

**DOI:** 10.1007/s44154-024-00162-0

**Published:** 2024-05-09

**Authors:** Hao Yu, Weimin Li, Xiaoxiao Liu, Qianqian Song, Junjun Li, Jin Xu

**Affiliations:** 1 College of Horticulture, Shanxi Agricultural University, Taigu, 030801 China; 2 Shanxi Key Laboratory of Germplasm Resources Innovation and Utilization of Vegetable and Flower, Taiyuan, 030031 China

**Keywords:** Nickel toxicity, Micronutrients, Oxidative stress response, Phytohormones, Transcription regulatory network

## Abstract

Nickel (Ni), a component of urease, is a micronutrient essential for plant growth and development, but excess Ni is toxic to plants. Tomato (*Solanum lycopersicum* L.) is one of the important vegetables worldwide. Excessive use of fertilizers and pesticides led to Ni contamination in agricultural soils, thus reducing yield and quality of tomatoes. However, the molecular regulatory mechanisms of Ni toxicity responses in tomato plants have largely not been elucidated. Here, we investigated the molecular mechanisms underlying the Ni toxicity response in tomato plants by physio-biochemical, transcriptomic and molecular regulatory network analyses. Ni toxicity repressed photosynthesis, induced the formation of brush-like lateral roots and interfered with micronutrient accumulation in tomato seedlings. Ni toxicity also induced reactive oxygen species accumulation and oxidative stress responses in plants. Furthermore, Ni toxicity reduced the phytohormone concentrations, including auxin, cytokinin and gibberellic acid, thereby retarding plant growth. Transcriptome analysis revealed that Ni toxicity altered the expression of genes involved in carbon/nitrogen metabolism pathways. Taken together, these results provide a theoretical basis for identifying key genes that could reduce excess Ni accumulation in tomato plants and are helpful for ensuring food safety and sustainable agricultural development.

**Supplementary Information:**

The online version contains supplementary material available at 10.1007/s44154-024-00162-0.

## Introduction

Nickel (Ni) is an essential trace element in plants; however, excess Ni is toxic to plant growth and development (Ghasemi et al. 2009). Ni toxicity inhibits plant growth by affecting photosynthesis, root elongation and nutrient uptake, thereby reducing crop yields (Hassan et al. 2019). Ni is one of the 23 metallic pollutants that make up 3% of the total composition of the earth (Duda-Chodak and Blaszczyk 2008). In recent years, with the acceleration of urbanization and industrialization, Ni toxicity has become a worldwide problem that threatens sustainable agricultural development (Yusuf et al. 2011; Pan et al. 2018). Ni deposition in agricultural soils occurs mainly through natural (wind and sand, volcanic eruptions, etc*.*) and anthropogenic activities (composting, increased use of greenhouses, low recycling of mulch, etc*.*) (Ameen et al. 2019; Xu et al. 2022). In China, a national survey showed that 4.8% of farmland soil was contaminated with Ni, making it the second most important soil pollutant (Zhao et al. 2015). As a mobile element, Ni can migrate from soil to edible parts of crops. Bioaccumulation of Ni in edible parts of crops increases food chain contamination (Cempel and Nikel 2006). Excessive exposure to Ni can lead to diseases such as cancer, and fibrosis of the lungs, posing a serious threat to human health (Genchi et al. 2020). Therefore, reducing the uptake of Ni by plants in Ni-enriched soils and increasing the tolerance of plants to Ni toxicity are highly important for ensuring ecosystem health and sustainable agricultural development.

The plant root system absorbs Ni in ionic form from the soil by active and passive migration (Ameen et al. 2019). Ni uptake is an active process via ZRT/IRT-like (ZIP) transporters and Natural resistance-associated macrophage proteins (NRAMPs) with low specificity in plants (Mizuno et al. 2005). In addition, members of the Mg^2+^-transporting MRS/MGT family in Arabidopsis exhibit Ni^2+^ uptake activity (Li et al. 2001). Ni translocation and accumulation are facilitated by binding to intracellular metal chelators, such as nicotinamide (NA), histidine (His) and organic acids (citric acid and malate). The maize yellow stripe-like (YSL) transporter ZmYS1 mediates long-distance transport of the Ni(II)-NA complex in plants (Schaaf et al. 2004). After Ni enters plants, it is usually stored in epidermal cells and vesicles rather than within the cell wall (Ahmad and Ashraf 2011). Arabidopsis IRON REGULATED 2 (IREG2), which is localized in the vacuolar membrane, is a core gene involved in the Ni toxicity response and in the transport of Ni into root vacuoles. Overexpression of *IREG2* results in increased Ni tolerance and increased Ni accumulation in roots (Schaaf et al. 2006).

Phytohormones play crucial roles in coordinating stress and growth to survive heavy metal toxicity (Saini et al. 2021; Cha et al. 2022; Bhat et al. 2023). The exogenous auxin indole-3-acetic acid (IAA) alleviates Ni toxicity, and overexpression of the auxin biosynthesis-related gene *YUC6* improves Ni toxicity tolerance by enhancing peroxidase (PRX) activity and reducing reactive oxygen species (ROS) accumulation through thiol-reductase (TR) activity in YUC6 in Arabidopsis (Cha et al. 2022). Gibberellic acid (GA) promotes Ni sequestration in vesicles and transport by upregulating the expression of *GmPC1* in soybean plants (Bhat et al. 2023); moreover, GA also upregulates the expression of *catalase* (*CAT*), *iron superoxide dismutase (Fe-SOD*), *ascorbate peroxidase (APX*) and *glutathione 1 (GSH1*), thus alleviating excess Ni-induced oxidative damage in soybean plants and ultimately improving yield (Bhat et al. 2023). Exogenous abscisic acid (ABA) can effectively reduce root Ni absorption and alleviate Ni-induced oxidative damage through the nitric oxide (NO) and hydrogen peroxide (H_2_O_2_) signaling pathways (Parwez et al. 2023). Inhibition of ethylene production improves Ni toxicity tolerance by reducing ROS overaccumulation (Khan and Khan 2014). JA (jasmonic acid) and SA (salicylic acid) both improve Ni toxicity tolerance by increasing the content of osmoregulatory substances and antioxidant enzyme activities (Wang et al. 2009; Sirhindi et al. 2016).

Tomato (*Solanum lycopersicum* L.) is one of the popular vegetables worldwide (Vats et al. 2022). Excessive use of fertilizers and pesticides, sewage irrigation and manure has resulted in Ni contamination in agricultural soils (Hassan et al. 2019; Roccotiello et al. 2022). However, the mechanisms underlying the Ni toxicity response in tomato plants have not been fully elucidated. In this study, we investigated the molecular regulatory network of tomato plants in response to Ni stress. Our results provide a theoretical basis for identifying key genes and signaling pathways that could reduce excess Ni accumulation in tomato plants and are helpful for ensuring food safety and sustainable agricultural development. These results obtained in this study provide a theoretical basis for an in-depth investigation of the adaptive mechanisms of tomatoes in response to Ni toxicity.

## Results

### Physiological effects of Ni toxicity on tomato seedling growth

**Fig. 1:**
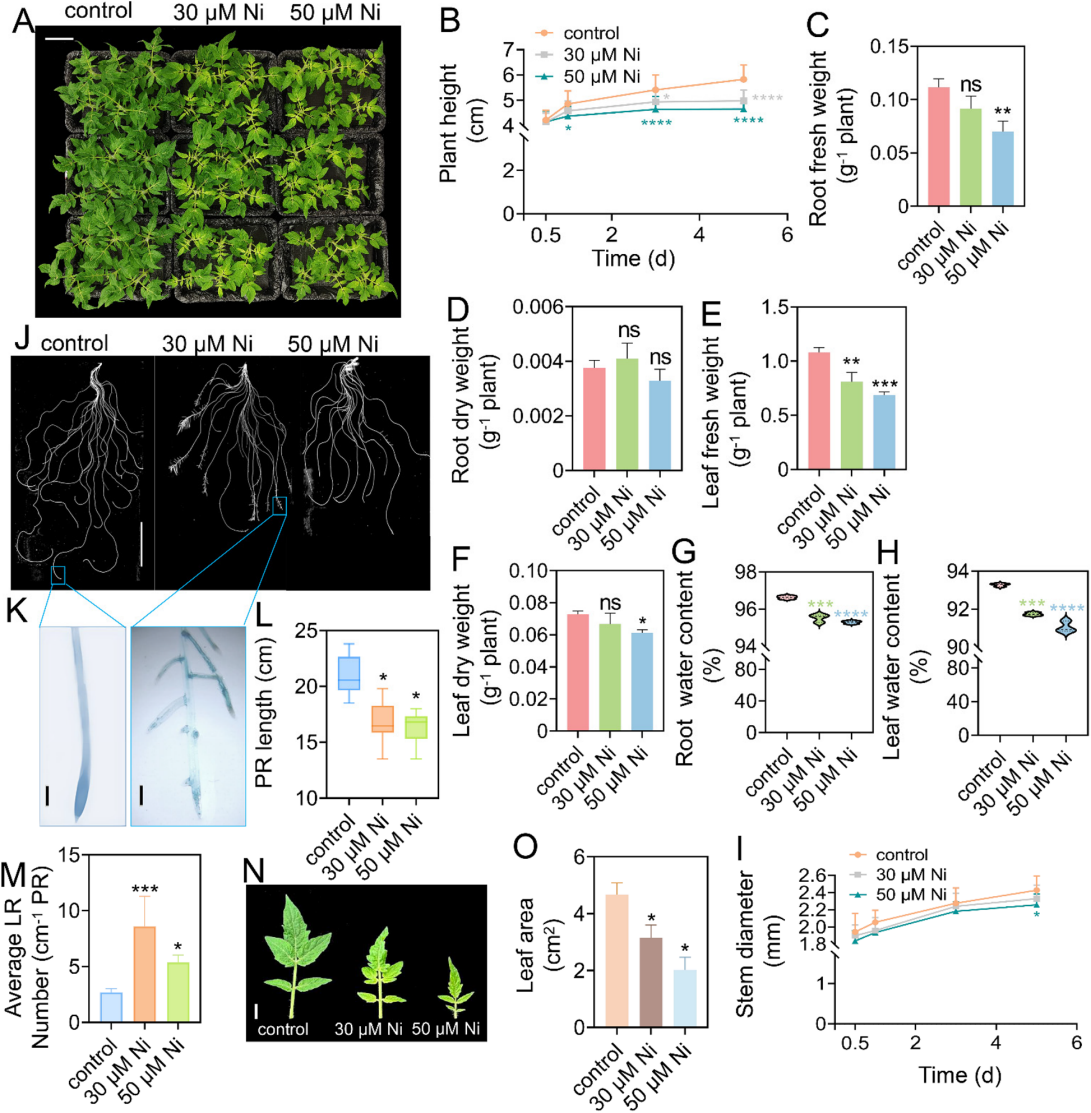
Nickel toxicity inhibited tomato seedling growth. Twenty-five-day-old tomato seedlings were transferred to fresh 1/4 Hoagland solution supplemented with or without 30 μM Ni or 50 μM Ni for 5 days. **A-I**, Representative images showing the plant phenotype (bar = 5 cm) (**A**), plant height (**B**), root fresh weight (FW) (**C**), root dry weight (DW) (**D**), leaf FW (**E**), leaf DW (**F**), root water content (**G**), leaf water content (**H**) and stem diameter (**I**) were measured. **J-O**, Representative images showing the root phenotype (bar = 5 cm) (**J**), the outgrowth of lateral roots (LRs) at the root tips (bar = 1 mm) (**K**), the primary root (PR) length (**L**) and the average LR number (**M**). **N** and** O**, Representative images showing the leaf phenotype (bar = 1 cm) (**N**), and the leaf area was measured (**O**). The values are given as the means ± SDs (*n* = 3, 6 seedlings/treatment), (* *P* < 0.05, **P < 0.01, ***P < 0.001, ****P < 0.0001; ANOVA)

Ni toxicity markedly inhibited plant growth (Fig. 1). The tomato plants exhibited severe dwarfing under Ni toxicity (Fig. 1A and B). Compared with those of the control, Ni toxicity reduced the fresh weight (FW), dry weight (DW) and the water contents of the leaves and roots of tomato plants (Fig. 1C-H). Ni toxicity also inhibits stem growth (Fig. 1I). Ni toxicity inhibited primary root (PR) growth but induced lateral root (LR) formation (Fig. 1J-M). Notably, Ni toxicity induced the formation of brush-like LRs in the region originally occupied by the mature zone of the root tips, especially under 30 μM Ni toxicity (Fig. 1K), suggesting that excess Ni leads to the premature differentiation of the root apical meristem in tomato plants. In addition, Ni toxicity also markedly inhibited leaf growth (Fig. 1N and O).

**Fig. 2:**
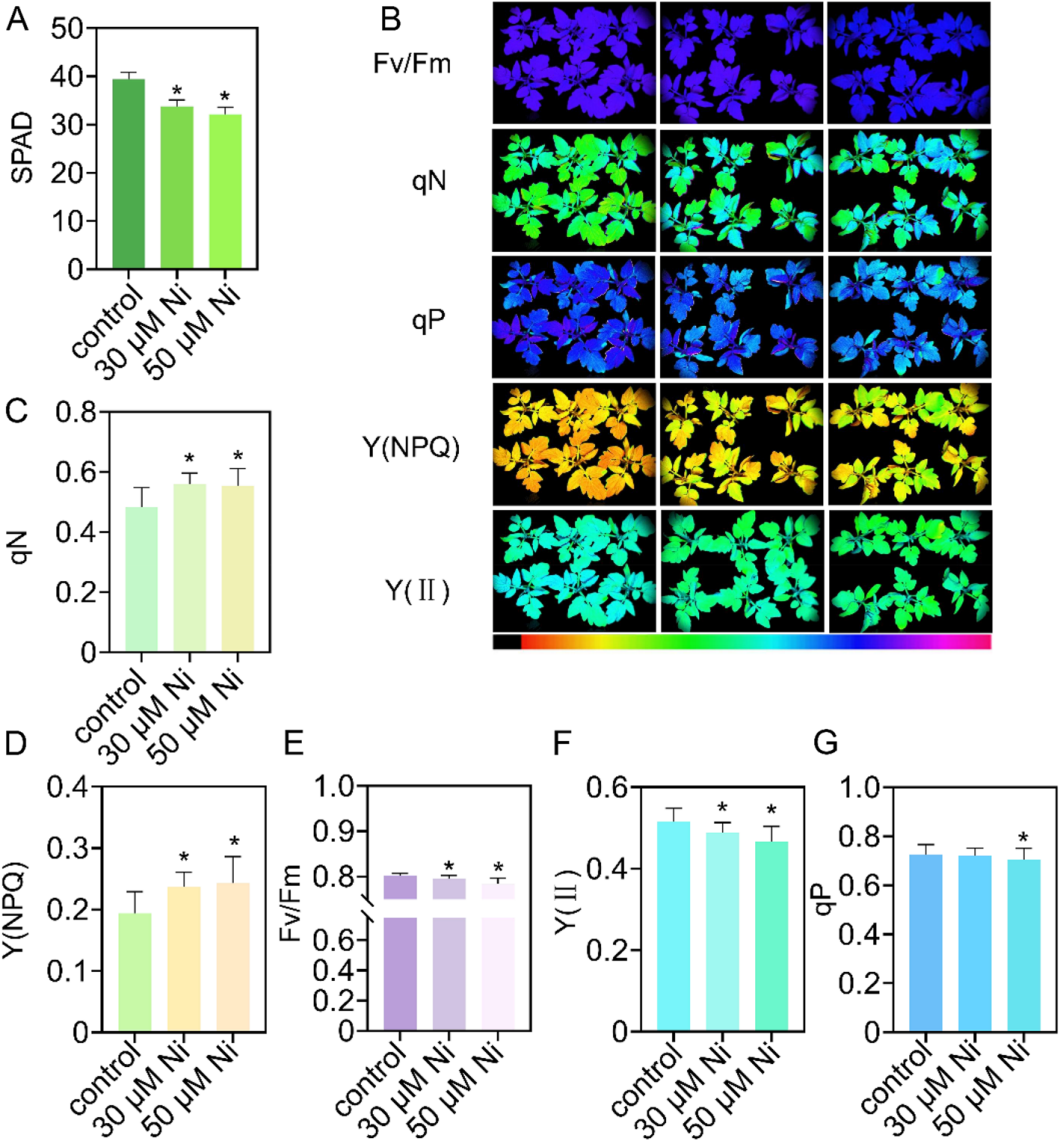
Effects of nickel toxicity on photosynthesis in tomato seedlings. Twenty-five-day-old tomato seedlings were transferred to fresh 1/4 Hoagland solution supplemented with or without 30 μM Ni or 50 μM Ni for 5 days. **A**, SPAD values. **B-G**, Representative images showing the chlorophyll fluorescence parameters (**B**) and the quantification of qN (**C**), Y (NPQ) (**D**), Fv/Fm (**E**), Y(II) (**F**) and qP (**G**). The values are given as the means ± SDs (*n* = 3, 6 seedlings/treatment). One-way analysis of variance (* *P* < 0.05, ANOVA)

Ni toxicity results in leaf chlorosis in tomato seedlings. Compared with those in the control, the chlorophyll contents in the 30 and 50 μM Ni treatment groups were reduced by 14.3% and 18.4%, respectively (Fig. 2A). Subsequently, we examined chlorophyll fluorescence in tomato leaves (Fig. 2B-G). Under 30 and 50 μM Ni treatments, qN increased by 15.9% and 15.0%, respectively (Fig. 2C); Y(NPQ) increased by 22.1% and 25.3%, respectively (Fig. 2D); Fv/Fm decreased by 0.8% and 2.3%, respectively (Fig. 2E); and Y(II) decreased by 5.0% and 9.2%, respectively (Fig. 2F). Furthermore, qP decreased by 3.1% under 50 μM Ni toxicity (Fig. 2G).

**Fig. 3:**
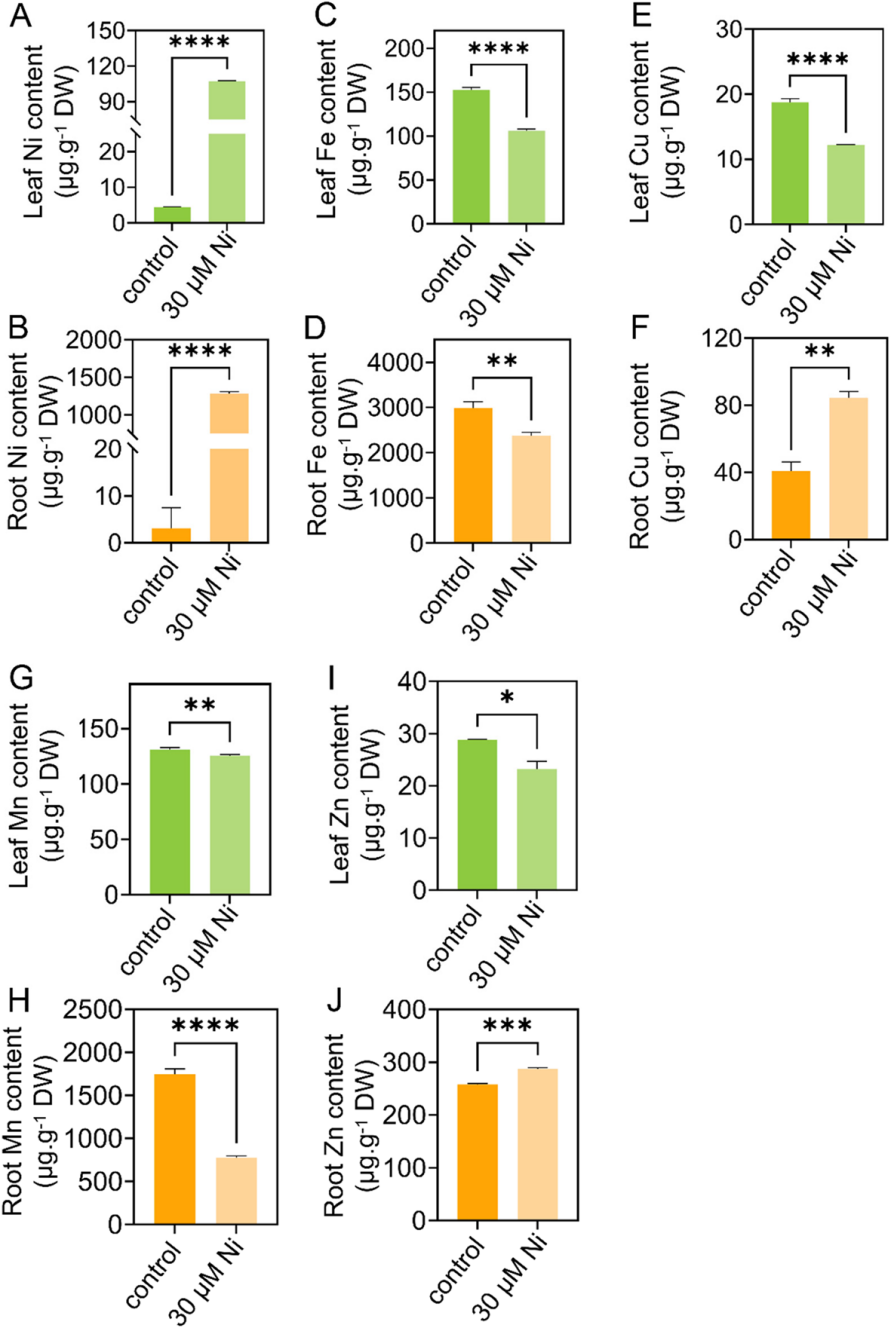
Nickel toxicity affects micronutrient element contents in tomato seedlings. **A-J**, Twenty-five-day-old tomato seedlings were transferred to fresh 1/4 Hoagland solution with or without 30 μM Ni for 5 d, the content of Ni in leaves (**A**) and roots (**B**), Fe in leaves (**C**) and roots (**D**), Cu in leaves (**E**) and roots (**F**), Mn in leaves (**G**) and roots (H), and Zn in leaves (**I**) and roots (**J**) were determined. The values are given as the means ± SDs (*n* = 3, 6 seedlings/treatment), (* *P* < 0.05, ** *P* < 0.01, *** *P* < 0.001, **** *P* < 0.0001, ANOVA)

We then examined the trace element contents in the tomato plants. After 30 μM Ni treatment, the Ni content in the leaves and roots increased by 23.5-fold and 41.2-fold, respectively, compared to that in the control (Fig. 3A and B). Under Ni stress, the Fe content decreased by 30.4% and 20.5% in the leaves and roots, respectively (Fig. 3C and D); the copper (Cu) content decreased by 34.8% in the leaves but increased by 1.07-fold in the roots (Fig. 3E and F); the manganese (Mn) content decreased by 4.2% and 55.5%, respectively, in the leaves and roots (Fig. 3G and H); and the zinc (Zn) content decreased by 19.5% in the leaves but increased by 11.5% in the roots (Fig. 3I and J).

### Transcriptome analysis

A transcriptome analysis was performed to detect the differentially expressed genes (DEGs) in the roots of tomato plants after 0, 4, 6, 12, or 24 h of 50 μM Ni treatment (Supplementary Fig. 1; Supplementary Table 1). A total of 94.94 Gb of clean data was obtained, and the percentage of Q30 bases in each sample was not less than 93.42%. Hierarchical clustering (Supplementary Fig. 1A) and intragroup correlation analysis were performed using Pearson’s correlation coefficient (Supplementary Fig. 1B), which revealed clear differences among the five treatment groups and good similarity among the three biological replicates in each group. A total of 2,713 DEGs were identified in Ni-4 h/control (1,775 upregulated and 938 downregulated genes), 1,804 DEGs were identified in Ni-6 h/control (1,248 upregulated genes, 556 downregulated genes), 1,690 DEGs were identified in Ni-12 h/control (767 upregulated, 923 downregulated genes), and 1,561 DEGs were identified in Ni-1d/control (800 upregulated, 761 downregulated genes) (false discovery rate (FDR) < 0.01 and log_2_ FC > 1 or < -1) (Supplementary Fig. 1C). We randomly selected seven genes to verify the accuracy of the transcriptome data, and the RT‒qPCR results showed good consistency between the RT‒qPCR and transcriptome data, indicating that the transcriptome data were reliable (Supplementary Fig. 1D and E).

We then performed weighted gene coexpression network analysis (WGCNA). A power value of 25 was selected as the optimal soft threshold in the network topology (Supplementary Fig. 2). A total of eight modules were generated from the WGCNA, and four significant modules were screened by correlation analysis between the modules and samples (correlation coefficient > 0.9, *P* < 0.05) (Supplementary Fig. 3A-E). Subsequently, a network diagram of the gene ontology (GO) enrichment analysis was constructed based on these significant modules. The four major regions were divided according to different biological functions in these modules, including metabolism, signaling pathways, response to the stimulus and transport (Supplementary Fig. 4). DEGs in the green module, which were involved mainly in “transport” and “response to stimulus”, were generally upregulated after Ni treatment; DEGs in the blue module, which were involved mainly in “hormone signaling”, “oxidative stress”, “metal ion transport”, “channel activity” and “nitrogen compounds”, were generally downregulated after Ni treatment; and DEGs in the brown module, which were involved mainly in “amino acid metabolism”, “signaling pathways” and “hormone signaling”, were continuously upregulated after Ni treatment, reaching a peak at 4 h and then downregulated. No significant GO enrichment category was obtained from the pink module (Supplementary Figs. 3 and 4).

### Ni toxicity alters the expression of genes involved in metal ion accumulation in roots

The above results showed that Ni toxicity affected the accumulation of trace elements (Fig. 3). GO enrichment analysis also revealed that Ni toxicity modulated metal ion transport (Supplementary Fig. 4). We thus investigated the genes involved in metal ion accumulation, and 25 DEGs were identified in the tomato roots (Supplementary Fig. 5; Supplementary Table 2). bHLH100 is an iron (Fe) deficiency-responsive transcription factor that upregulates the expression of genes related to Fe uptake and accumulation, such as *iron-regulated transporter 1* (*IRT1*) and *FRO2*, in plants (Hirayama et al. 2018; Wang et al. 2020). Ni toxicity downregulated *bHLH100-like* (*bHLH100L*) expression (Supplementary Fig. 5). Moreover, the expression of *FRO1* and *FRO2* was also significantly downregulated after Ni treatment (Supplementary Fig. 5). Vacuolar iron transfer proteins (VITs) are responsible for Fe storage in vacuoles (Cao 2019). NRAMPs modulate the uptake and compartmentalization of divalent ions such as Fe^2+^, Mn^2+^, Cu^2+^, Zn^2+^, Cd^2+^ and Ni^2+^ in plants (Cun et al. 2014). Metal tolerance proteins (MTPs) mediate ionic homeostasis by regulating the uptake of Zn^2+^, Fe^2+^, Co^2+^, Ni^2+^, Cd^2+^ and Mn^2+^ in plants (Socha and Guerinot 2014). Similarly, the expression of *NRAMP1*, five *VIT* and three *MTP* genes was significantly downregulated after Ni treatment (Supplementary Fig. 5). bZIP23 is a Zn sensor that modulates Zn uptake in cells by inducing the expression of *ZIPs* (Lilay et al. 2019). Ni toxicity upregulated *bZIP23* expression (Supplementary Fig. 5). Furthermore, the expression of the three *ZIP* genes was also significantly upregulated after Ni treatment (Supplementary Fig. 5). IREG3 is involved in Fe export to mitochondria (Kim et al. 2021). The metal-nicotianamine transporter YSLs play a role in the long-distance transport of metal ions (Curie et al. 2009). The copper transporters (CTRs) are involved in Cu ion uptake (Vatansever et al. 2017). The expression of the *IREG3*, *YSL2* and two *CTRs* was also significantly upregulated after Ni treatment (Supplementary Fig. 5).

### Ni toxicity affects phytohormone levels and the expression of genes involved in phytohormone signaling pathways

**Fig. 4:**
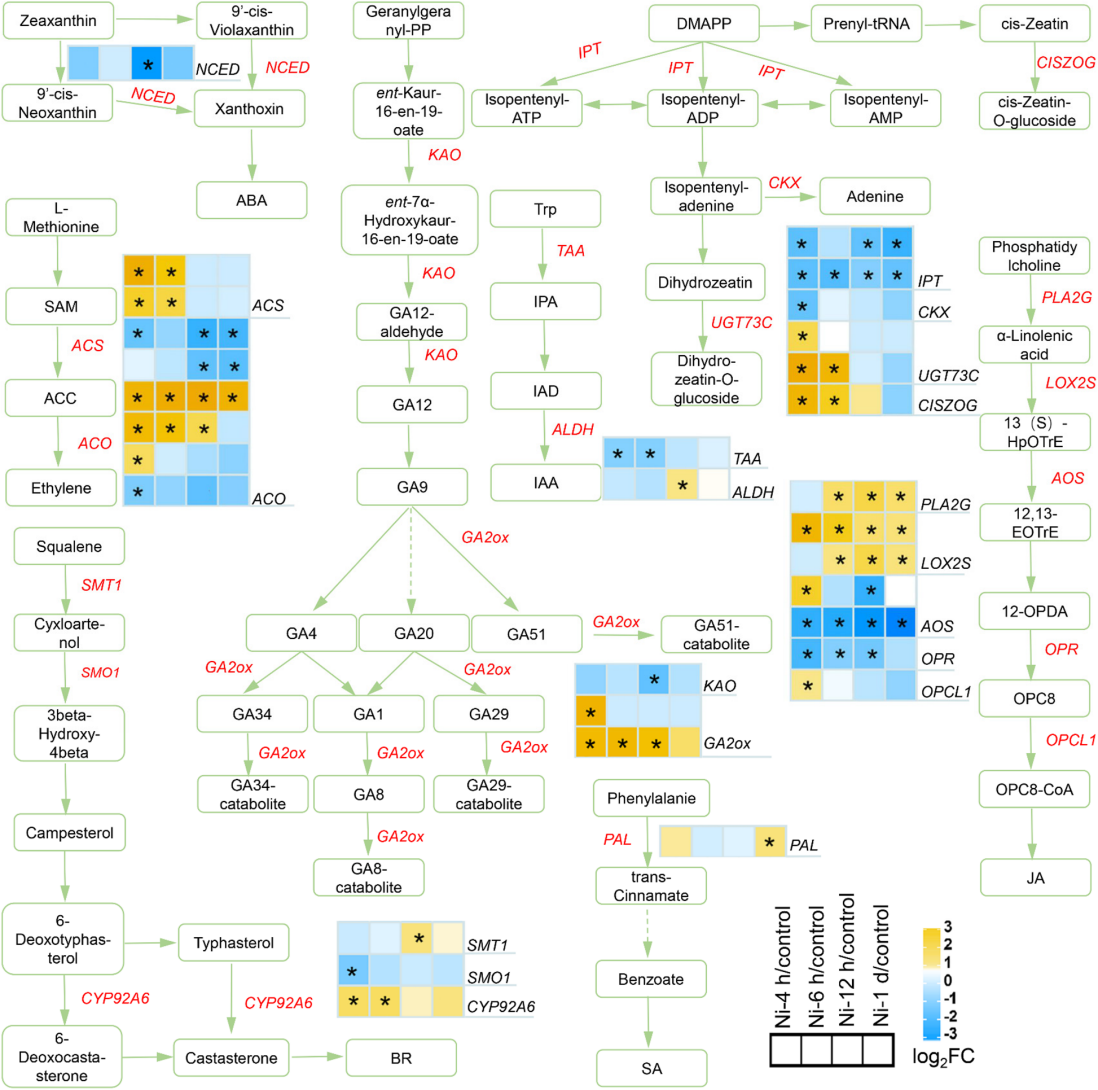
Expression of genes related to phytohormone biosynthesis in tomato roots in response to excess Ni. The heatmaps show the gene expression patterns according to the log_2_(fold change), and the asterisks in the heatmaps represent the DEGs

GO enrichment analysis revealed that several pathways involved in the response to phytohormones were enriched in the roots of the Ni-treated tomato plants (Supplementary Fig. 4). Therefore, we investigated DEGs associated with phytohormone biosynthesis (Fig. 4; Supplementary Table 3). Ni toxicity downregulated the expression of *9-cis-epoxycarotenoid dioxygenase* (*NCED*), a key ABA biosynthesis gene, whereas it upregulated the expression of *phenylalanine ammonia-lyase* (*PAL*), a SA biosynthesis-related gene (Fig. 4). *ACC synthase* (*ACS*) and *ACC oxidase* (*ACO*) are the key genes involved in the biosynthesis of the ethylene precursor 1-aminocyclopropane-1-carboxylic acid (ACC) and ethylene in plants (Houben and Van de Poel 2019). The expression of two *ACS* genes and three *ACO* genes was upregulated, whereas the other three *ACO* genes were downregulated (Fig. 4). In the brassinolide (BR) biosynthesis pathway, the expression of one *sterol-C24-methyltransferase 1* (*SMT1*) gene and one *cytochrome P450 CYP92A6* gene was upregulated (Fig. 4). In the GA biosynthesis pathway, the expression of *ent-kaurenoic acid monooxygenase* (*KAO*) was downregulated, whereas two *gibberellin 2 beta-dioxygenase* (*GA2ox*) genes involved in the deactivation of bioactive GAs were upregulated (Fig. 4). *Tryptophan aminotransferase* (*TAA*) and *aldehyde dehydrogenase (NAD* +) (*ALDH*) are the key genes involved in IAA biosynthesis. The expression of one *TAA* gene was downregulated, whereas one *ALDH* gene was upregulated (Fig. 4). In the cytokinin biosynthesis pathway, the expression of two *adenylate dimethylallyl transferase* (*IPT*) genes involved in cytokinin biosynthesis and one *cytokinin dehydrogenase* (*CKX*) gene involved in irreversible degradation of cytokinin was downregulated, while the expression of one *cis-zeatin O-glucosyl transferase* (*CISZOG*) and two *glucosyltransferase 73C* (*UGT73C*) involved in inactivation of cytokinin was upregulated (Fig. 4). In the JA biosynthesis pathway, the expression of one *OPC-8:0 CoA ligase 1* (*OPCL1*), one *secretory phospholipase A2* (*PLA2G*) and two *lipoxygenase 2S* (*LOX2S*) was upregulated, whereas one *hydroperoxide dehydratase* (*AOS*) and one *12-oxophytodienoic acid reductase* (*OPR*) gene were downregulated by Ni toxicity (Fig. 4).

**Fig. 5:**
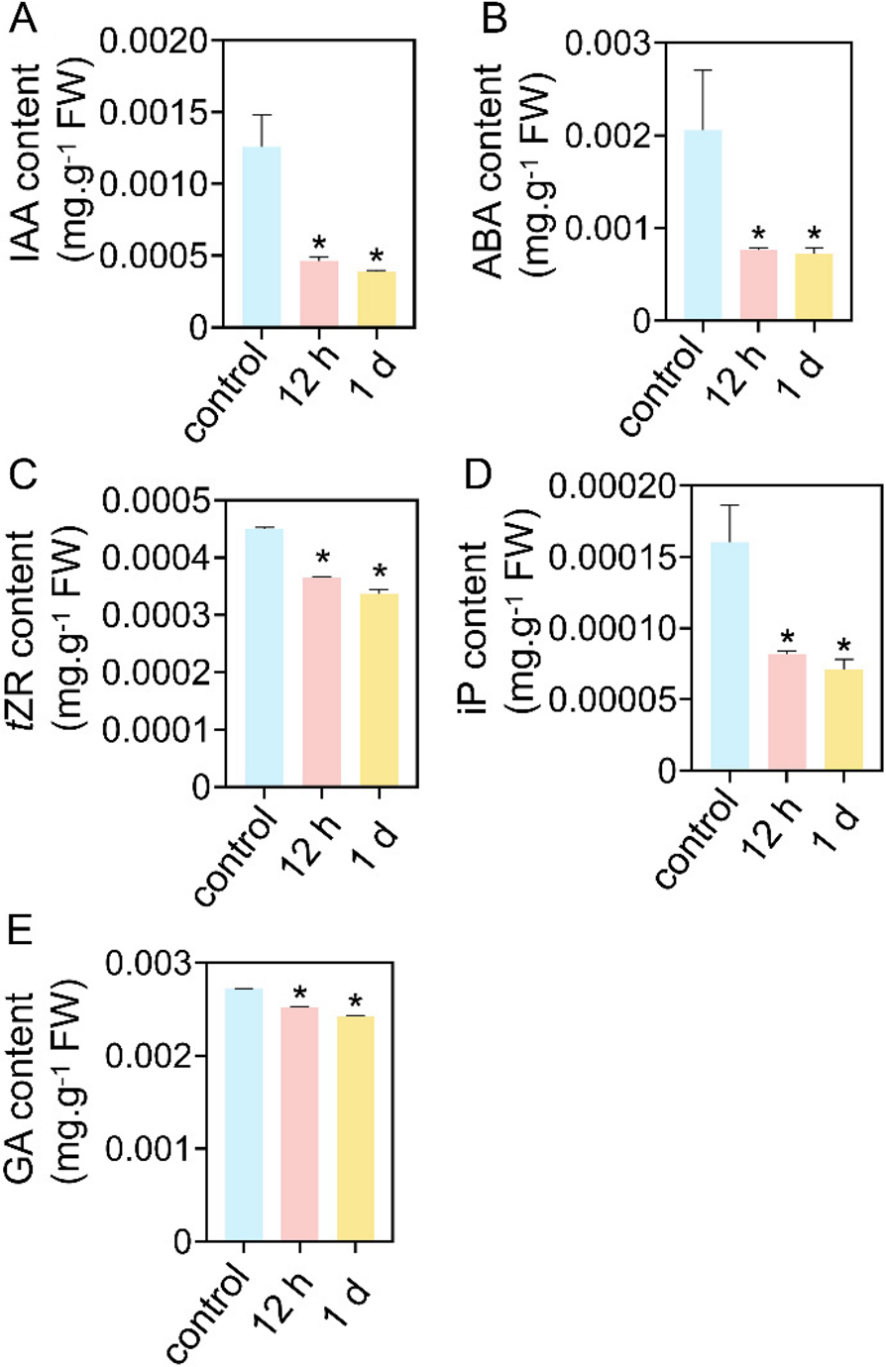
Ni toxicity affects phytohormone levels in the roots of tomato seedlings. Twenty-five-day-old tomato seedlings were transferred to fresh 1/4 Hoagland solution supplemented with or without 50 μM Ni for 12 h or 1 d, after which the contents of IAA (**A**), ABA (**B**), *t*ZR (**C**), iP (**D**) and GA (**E**) were determined. The values are given as the means ± SDs (*n* = 3, 6 seedlings/treatment), (* *P* < 0.05, ANOVA)

We subsequently determined the phytohormone contents in the roots of the Ni-treated tomato plants. As shown in Fig. 5A-E, after 12 h of Ni treatment, the levels of IAA, ABA, GA and the two cytokinins *trans*-zeatin riboside (*t*ZR) and isopentenyl-adenine (iP) decreased by 63.1%, 62.8%, 7.3%, 19.1% and 49%, respectively, while they decreased by 69.0%, 65.0%, 11.0%, 25.2% and 55.6%, respectively, after 24 h of Ni treatment.

**Fig. 6:**
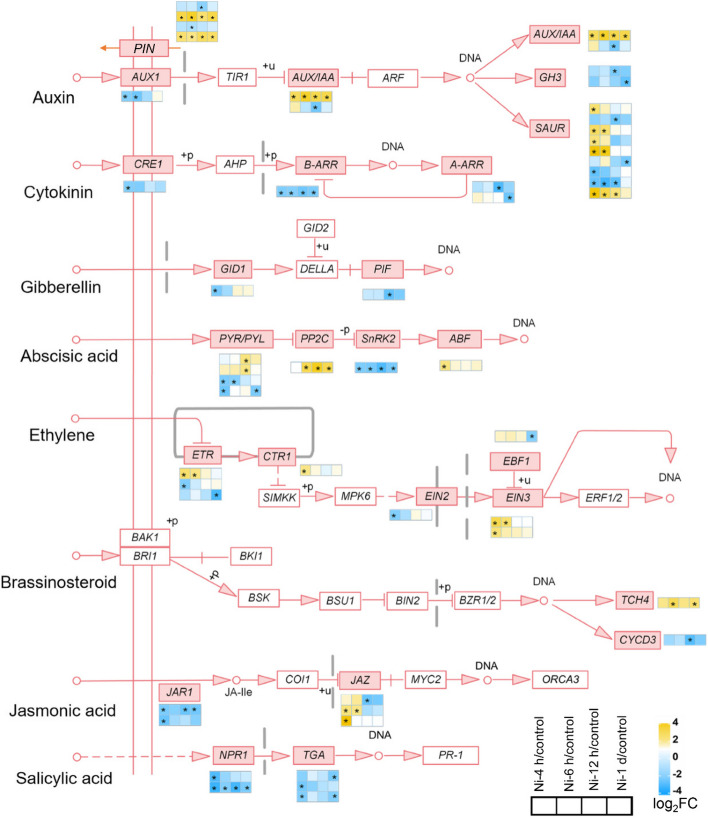
Expression of genes involved in phytohormone signaling pathways in tomato roots in response to excess Ni. The heatmaps show the gene expression patterns according to the log_2_(fold change), and the asterisks in the heatmaps represent the DEGs

Subsequently, we investigated the expression of DEGs involved in phytohormone signaling pathways (Fig. 6; Supplementary Table 4). In the auxin pathway, the expression of two *GH3* genes, one auxin influx carrier *AUXIN 1* (*AUX1*), two *PIN-FORMED* (*PIN*) auxin exporters *PIN4* and *PIN9*, one *AUX/IAA* and four *SAUR* genes was downregulated, whereas two *PIN* genes (*PIN5* and *PIN10*), one *AUX/IAA* gene and five *SAUR* genes was upregulated. Ni toxicity inhibits the cytokinin, GA, JA and SA signaling pathways in the roots of Ni-treated tomatoes. In the cytokinin pathway, the expression of two *A-ARR* genes, one B-type response regulator (*B-ARR*) and one *cytokinin response 1* (*CRE1*) was downregulated. In the GA pathway, the expression of one GA receptor gene, *gibberellin insensitive dwarf 1* (*GID1*), and one GA-responsive gene, *phytochrome-interacting factor* (*PIF*), was downregulated. In the JA pathway, the expression of two *jasmonate resistant 1* (*JAR1*) genes involved in the biosynthesis of JA-Ile, and one *jasmonate ZIM-domain* (*JAZ*) gene, which is the repressor of JA signaling, was downregulated, whereas the other two *JAZ* genes were upregulated. In the SA pathway, the expression of three *TGA* and two *NPR1* was downregulated. In addition, Ni toxicity affects the signaling pathways of ABA, ethylene and BR. In the ABA pathway, the expression of one *ABRE-binding factor* (*ABF*), one *protein phosphatase 2C* (*PP2C*) and two *pyrabactin resistance/PYR-like* (*PYR/PYC*) was upregulated, whereas the expression of two other *PYR/PYC* genes and one core component of ABA signaling, the *sucrose nonfermenting-1-related protein kinase 2* (*SnRK2*) gene, was downregulated. In the ethylene pathway, the expression of one positive regulator of one *EIN2*, two *ETR* genes and one *EBF1* gene was downregulated, whereas the other one ethylene receptor *ethylene response* (*ETR*), one negative regulator of ethylene signaling *constitutive triple response 1* (*CTR1*) and two positive regulators of ethylene signaling *EIN3* genes was upregulated. In the BR pathway, the expression of two downstream BR-responsive genes, *cyclin D3* (*CYCD3*) and *xyloglucan:xyloglucosyl transferase* (*TCH4*), was downregulated and upregulated, respectively (Fig. 6).

### Ni toxicity induces oxidative stress response

**Fig. 7:**
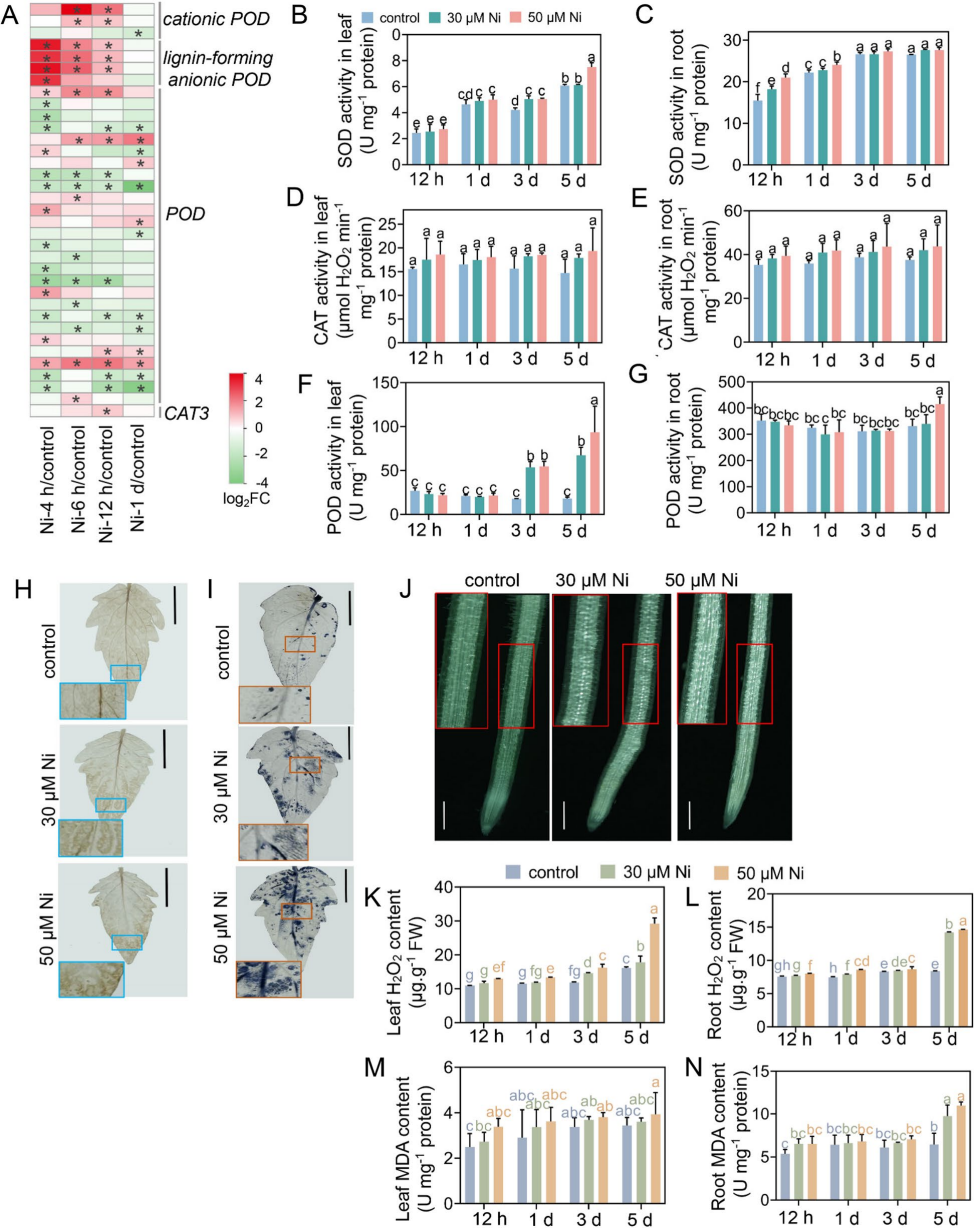
Nickel toxicity induced oxidative damage and affected antioxidant enzyme activities in tomato seedlings. **A**, Heatmaps showing the expression patterns of antioxidative enzyme-encoding genes according to the log_2_-fold change, and the asterisks in the heatmaps represent the DEGs. **B-G**, Twenty-five-day-old tomato seedlings were transferred to fresh 1/4 Hoagland solution supplemented with or without 30 μM Ni or 50 μM Ni for 12 h, 1 d, 3 d, or 5 d, after which the leaf superoxide dismutase (SOD) activity (**B**), root SOD activity (**C**), leaf catalase (CAT) activity (**D**), root CAT activity (**E**), leaf peroxidase (POD) activity (**F**) and root POD activity (**G**) were determined. The values are given as the means ± SDs. Different letters indicate significant differences (*P* < 0.05). **H-N**, Twenty-five-day-old tomato seedlings were transferred to fresh 1/4 Hoagland nutrient solution supplemented with or without 30 μM Ni or 50 μM Ni for 5 days. **H**, DAB staining. **I**, NBT staining. Bar = 1 cm. **J**, DCFH-DA fluorescence staining showing ROS levels in the root tips (bar = 500 μm). **K-N**, Twenty-five-day-old tomato seedlings were transferred to fresh 1/4 Hoagland nutrient solution supplemented with or without 30 μM Ni or 50 μM Ni for 12 h, 1 d, 3 d, or 5 d, after which the H_2_O_2_ (**K, L**) and MDA (**M, N**) contents in the leaves (**K, M**) and roots (**L, N**) were determined. The values are given as the means ± SDs (*n* = 3, 6 seedlings/treatment). Different letters indicate significant differences (*P* < 0.05, ANOVA)

GO enrichment analysis revealed that Ni toxicity induced oxidative stress responses in tomato roots (Supplementary Fig. 4). Therefore, we investigated the DEGs associated with the antioxidant system (Fig. 7A; Supplementary Table 5). Ni toxicity altered the expression patterns of the *peroxidase* (*POD*) and *catalase* (*CAT*) genes (Fig. 7A). The expression of the *CAT3* gene was upregulated after 12 h of Ni treatment. Ni toxicity also markedly induces the expression of *lignin-forming anionic POD* genes and *cationic POD* genes, thereby regulating cell wall metabolism and root system growth in response to excess Ni (Tamás et al. 2007). Furthermore, after excess Ni treatment, 50% of the *POD* genes were upregulated, whereas 47% of the *POD* genes were downregulated in the tomato roots (Fig. 7A).

We then examined antioxidative enzyme activities in tomato plants. Ni stress increased the activities of superoxide dismutase (SOD) and peroxidase (POD) both in leaves and roots; however, it did not significantly affect catalase (CAT) activity (Fig. 7B-G). Next, we detected ROS levels in tomato seedlings under Ni toxicity. 3,3'-diaminobenzidine (DAB) and nitroblue tetrazolium (NBT) staining further revealed that Ni toxicity induced H_2_O_2_ and O_2_^.−^ accumulation, respectively, in the leaves (Fig. 7H and I). Visualization of ROS levels by a 2,7-dichlorofluorescein diacetate (DCFH-DA) fluorescence probe showed that Ni toxicity induced ROS accumulation in roots (Fig. 7J). The quantitative detection of H_2_O_2_ content also confirmed these results (Fig. 7K and L). The malondialdehyde (MDA) content reflects the degree of oxidative damage in plants (Hodges et al. 1999). Ni toxicity did not significantly induce MDA accumulation in the leaves (Fig. 7M). However, the root MDA content increased by 51.1% and 70% after 5 days of 30 and 50 μM Ni treatment, respectively (Fig. 7N).

### Ni toxicity alters the expression of genes associated with the primary metabolism

**Fig. 8:**
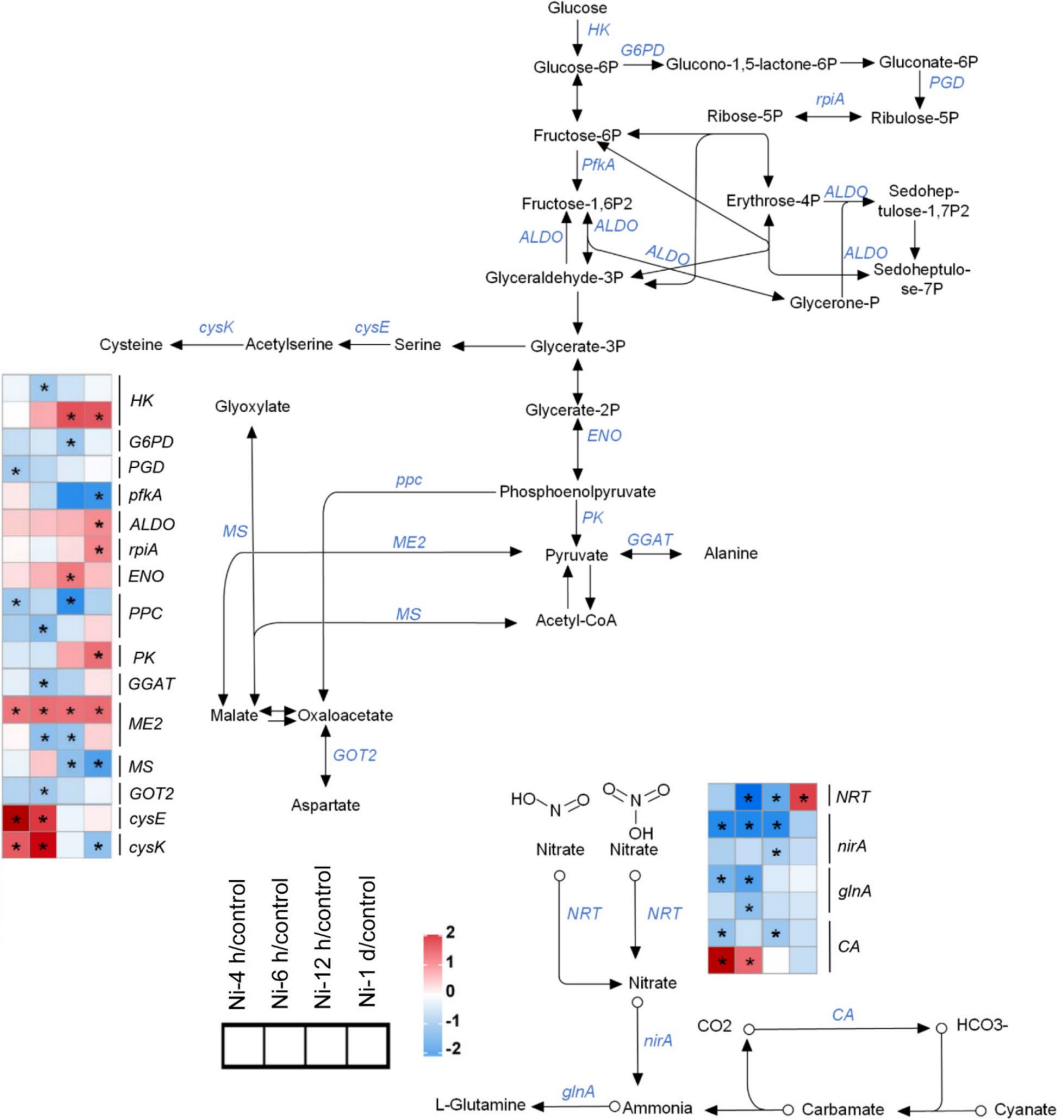
Expression of genes associated with carbon and nitrogen metabolism pathways in tomato roots in response to excess Ni. The heatmaps show the gene expression patterns according to the log_2_(fold change), and the asterisks in the heatmaps represent the DEGs

GO enrichment analysis revealed that Ni toxicity altered metabolic processes in tomato roots (Supplementary Fig. 4). Therefore, we analyzed the DEGs involved in carbon and primary nitrogen metabolism (Fig. 8; Supplementary Table 6). In glycolysis pathway, the expression of one *hexokinase* (*HK*), one *fructose-bisphosphate aldolase* (*ALDO*), one *enolase* (*ENO*) and one *pyruvate kinase* (*PK*) gene was upregulated under Ni toxicity, but one *HK* and one *6-phosphofructokinase A* (*pfkA*) gene were downregulated (Fig. 8). In the pentose phosphate pathway, the expression of one *G6PD* gene and one *6-phosphogluconate dehydrogenase* (*PGD*) gene was downregulated, whereas one *ribose 5-phosphate isomerase A* (*rpiA*) gene was upregulated (Fig. 8). In the glyoxylate and C4-dicarboxylic acid cycle pathway, the expression of one *glutamic-oxaloacetic transaminase 2* (*GOT2*), one *malate synthase* (*MS*), one *malate dehydrogenase* (*ME2*), one *glutamate: glyoxylate aminotransferase* (*GGAT*) and two *phosphoenolpyruvate carboxylase* (*PPC*) genes was downregulated, whereas one *ME2* gene was upregulated (Fig. 8). In the biosynthetic pathway of cysteine, the expression of one *serine acetyltransferase* (*cysE*) gene was upregulated after Ni treatment, whereas the expression of one *cysteine synthase* (*cysK*) gene was upregulated after 4 and 6 h of Ni treatment but downregulated after 1 d of Ni treatment (Fig. 8). In the primary nitrogen metabolism pathway, the expression of two *ferredoxin-nitrite reductases* (*nirA*), two *glutamine synthetases* (*glnA*) and one *carbonic anhydrase* (*CA*) gene was downregulated, whereas one *CA* gene was upregulated, and one *high-affinity nitrate transporter* (*NRT*) gene was downregulated after 6 and 12 h of Ni treatment and subsequently upregulated after 1 d of Ni treatment (Fig. 8).

## Discussion

Excess Ni inhibits growth and development and reduces yield and quality in crops (Ameen et al. 2019). Our study also indicated that excess Ni inhibits plant growth by decreasing the water content, chlorophyll level and PSII activity (Figs. 1 and 2). Ni toxicity inhibits root system growth and reduces the number of LRs in rice (Seregin et al. 2003; Rizwan et al. 2022), whereas it increases the density of LRs in Arabidopsis (Leškovï et al. 2020). We found that although excess Ni (30 or 50 μM) inhibited PR growth, it markedly induced LR formation and the formation of brush-like LRs at the root tips in tomato plants (Fig. 1J-M). This difference suggested that there were different response mechanisms involved in modulating root system growth in response to Ni toxicity between dicotyledons and monocotyledons. Increased LRs improve the absorption of water and nutrients, which is beneficial for enhancing plant tolerance to heavy metal toxicity. Therefore, this may be an adaptative mechanism for Arabidopsis and tomato plants in response to Ni toxicity. However, the underlying molecular mechanisms still need further clarification. Heavy metal toxicity interferes with the carbon and nitrogen supplies in plants (Ghori et al. 2019). A previous study demonstrated that Ni toxicity affects carbon and nitrogen metabolism in wheat (Gajewska and Skłodowska 2009; Gajewska et al. 2013). In support of these results, our results indicated that Ni toxicity altered the expression of genes involved in primary carbon and nitrogen metabolism (Fig. 8), ultimately modulating the adaptation of tomato plants to Ni toxicity.

Ni toxicity induced the expression of divalent metal cation long-distance transporter *YSL2*, metal transmembrane transporter *NRAMP3-Like* and divalent ion transporters *ZIPs* in roots (Mizuno et al. 2005; Oomen et al. 2009; Chu 2010) (Supplementary Fig. 5), thereby maintaining the uptake and accumulation of metal micronutrients in plants (Fig. 3A and B). Ni toxicity also upregulates the expression of two Cu transporter *CTRs* in roots. Consistent with these results, the Cu concentration in the roots was elevated in the Ni-treated tomato plants (Fig. 3F). FRO is responsible for Fe reduction from Fe^3+^ to Fe^2+^ (Bernal et al. 2012); subsequently, IRT1 transports Fe^2+^ into roots (Brumbarova et al. 2015). The Fe deficiency-responsive *bHLH100-like* gene encodes a transcription factor that directly upregulates the expression of *IRT1* and *FRO2*, thereby positively regulating Fe uptake. Ni toxicity downregulated the expression of *bHLH100-like*, *FRO1/2* and *IRT1* in roots (Supplementary Fig. 5); consistent with these results, the Fe levels in the leaves and roots of the Ni-treated plants were reduced (Fig. 3C and D). *VITs* are excess Fe-responsive genes that are involved in Fe compartmentalization in vacuoles (Kim et al. 2006; Peng and Gong 2014); moreover, Ni toxicity decreases Fe accumulation; therefore, *VIT* expression was downregulated in Ni-treated tomato roots (Fig. 3C and D; Supplementary Fig. 5). Ni toxicity induced the Zn sensor *bZIP23* expression, and three *ZIP* genes was also induced in the roots (Supplementary Fig. 5). Consistent with this result, Ni toxicity increased Zn accumulation in roots (Fig. 3J). Fe, Cu and Mn are essential components for maintaining chlorophyll structure and activity (Ahmad and Ashraf 2011). Ni toxicity reduces the levels of Fe, Cu and Mn in leaves (Fig. 3C, E and G), thereby repressing photosynthetic efficiency in tomato plants (Fig. 2).

Ni toxicity induces ROS overproduction in tomato plants (Fig. 7H-L). This result is consistent with previous reports that Ni toxicity induces oxidative damage in plants (Gajewska et al. 2006; Gajewska and Skłodowska 2007). ROS accumulation in roots promotes LR formation (Orman-Ligeza et al. 2016). Indeed, we observed brush-like LRs in the root tips of the Ni-treated tomato plants (Fig. 1J-M). Antioxidative enzymes play an important role in maintaining ROS homeostasis in vivo and preventing oxidative damage in plants under abiotic stresses (Dubey and Pandey 2011). Ni toxicity upregulated the expression of several *POD* genes and increased POD activity in tomato plants (Fig. 7A, F and G). Moreover, Ni toxicity also increased SOD activity in tomato plants (Fig. 7B and C) but did not affect the expression of *SOD* genes in roots. In addition, Ni toxicity upregulated *CAT3* expression in roots (Fig. 7A) but did not alter CAT activity (Fig. 7D and E). These results collectively indicated that Ni toxicity modulates antioxidative enzyme activities at both the transcriptional and posttranscriptional levels.

In this study, we found that excess Ni decreased the concentrations of auxin, cytokinin and GA in tomato roots (Fig. 5A-E), thereby slowing plant growth. Leškovï et al. (2020) reported that a high concentration of Ni (100 and 150 μM) inhibits PR growth by repressing *PIN2* abundance in root tips. We found that Ni toxicity (50 μM) upregulated the expression of *PIN5* and *PIN10* but downregulated the expression of *PIN4*, *PIN9* and *AUX1* in roots (Fig. 6), suggesting that excess Ni interferes with root auxin transport and distribution, thus modulating root system architecture. However, the detailed molecular mechanisms need to be further elucidated. Excess Ni downregulated *IPT* gene expression (Fig. 4). In support of these results, the contents of *t*ZR and iP decreased in the roots of the Ni-treated plants (Fig. 5C-D). In addition, Ni toxicity also upregulated the expression of the GA-inactivating enzyme *GA2ox*; consistent with these results, the GA content decreased in the roots (Figs. 4 and 5E). ABA plays important roles in modulating abiotic stress tolerance in plants. However, we found that Ni toxicity reduces ABA levels in roots (Fig. 5). Moreover, Ni toxicity upregulated the expression of *PP2C* genes, which are negative regulators of ABA signaling, while it downregulated the expression of ABA biosynthesis-related *NCED* genes, as well as the *SnRK2* gene, the core component of ABA signaling, thereby potentially inhibiting the ABA signaling pathway (Figs. 4 and 6). However, the detailed molecular mechanism by which excess Ni represses ABA levels and the ABA signaling pathway in tomato roots still need to be further elucidated.

## Conclusion

**Fig. 9:**
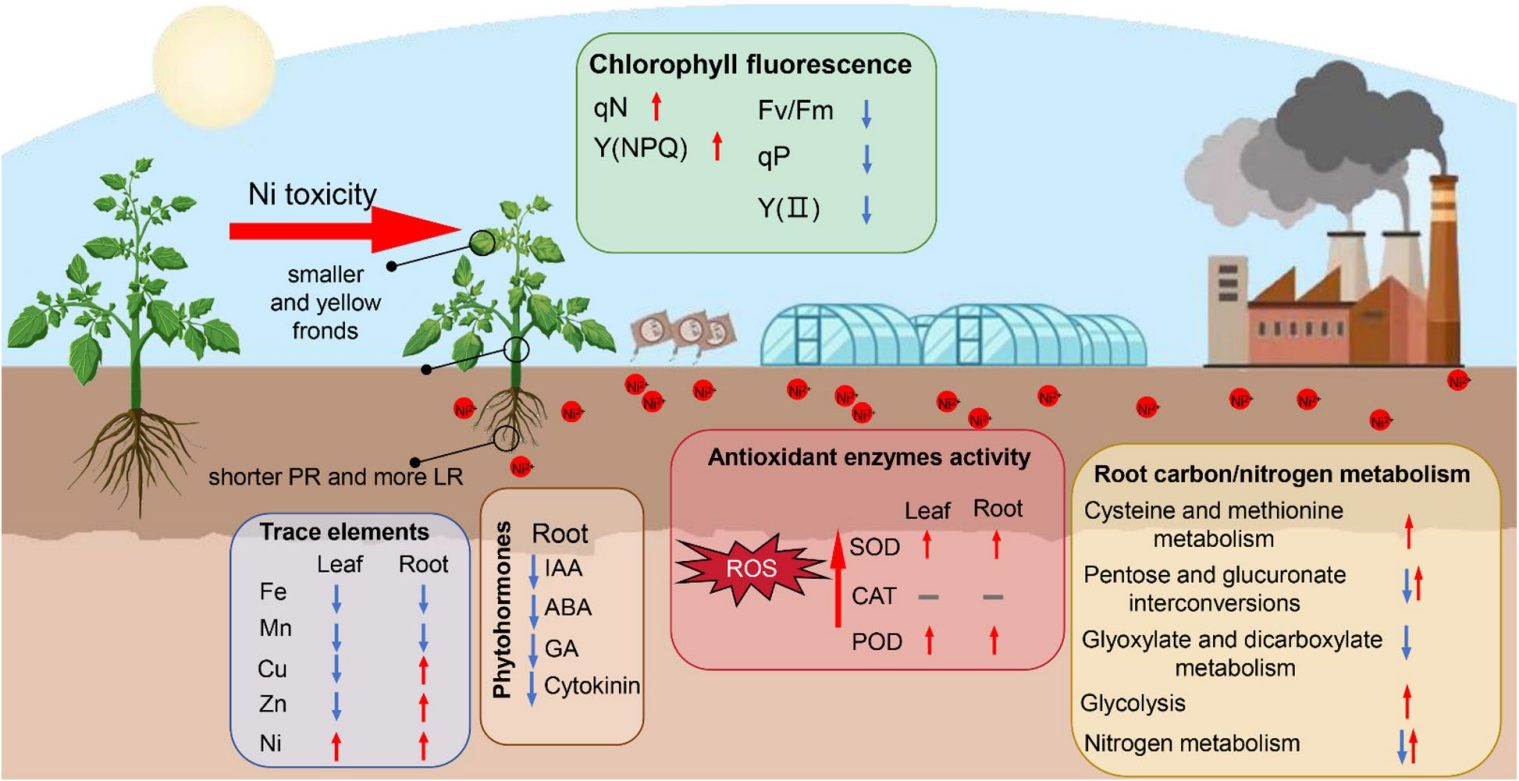
A proposed model for the Ni toxicity responses in tomato seedlings

In summary, this study investigated the physiological and molecular mechanisms underlying excess Ni-mediated growth in tomato plants via physio-biochemical and transcriptomic analyses. The results indicated that (i) excess Ni reprogrammed root system architecture by inducing the formation of brush-like LRs in tomato plants; (ii) excess Ni interfered with micronutrient accumulation and photosynthesis efficiency; (iii) excess Ni altered the expression of genes involved in primary metabolic processes; (iv) excess Ni reduced the levels of IAA, cytokinin and GA, ultimately maintaining tomato plant survival under Ni toxicity (Fig. 9). This work provides a basis for future in-depth studies of the molecular mechanisms involved in the Ni toxicity response in tomato plants.

## Materials and methods

### Plant materials and growth conditions

Tomato (*Solanum lycopersicum* L.) *cv.* micro-Tom seeds were soaked in sterile water for 30 min, surface sterilized with 75% (v/v) alcohol for 40 s, washed with 50% (v/v) bleach for 6 min, and then rinsed 5 times with sterile water. The sterilized seeds were placed on sprouting trays for germination. Twelve-day-old tomato plants were subsequently transferred to 1/4 Hoagland nutrient solution for 10 days. Twenty-five-day-old tomato plants were subsequently transferred to fresh 1/4 Hoagland solution supplemented with or without 30 or 50 μM NiCl_2_ for 5 days.

### Phenotypic analysis

The plant stem thickness was measured with a Vernier caliper. The root and leaf images were obtained by scanning with an Epson Perfection V500 Photo scanner (Epson, Japan), and ImageJ (version 1, 44) software was subsequently used to measure PR length, plant height and leaf area. The FW and DW were measured, and the plant water content was calculated using the following formula: plant water content (%) = (FW-DW)/DW.

The roots were immersed in FAA fixative (5 ml of 38% formaldehyde, 5 ml of glacial acetic acid, 90 ml of 50% alcohol, and 5 ml of glycerol) and fixed for more than 24 h. The fixed roots were first rehydrated by a gradient of 50% ethanol and 30% ethanol for 5 min, soaked in distilled water for 10 min, and then transferred to 0.01% methylene blue staining solution for 7–10 min. The roots were washed three times with distilled water, and the number of LRs was observed and counted using an optical microscope (Leica, Germany).

### Determination of chlorophyll contents and photosynthetic indices

The chlorophyll contents were determined using a SPAD-502 chlorophyll meter (Minolta Camera Co., Ltd., Japan). Fluorescence detection was performed using a MAXI imaging PAM instrument (Heinz Walz GmbH, 91,090 Effeltrich, Germany). The seedlings were pretreated in the dark for 30 min and subsequently placed in the instrument to determine the chlorophyll fluorescence parameters. The fluorescence parameters were calculated as follows: PS II maximum activity parameter Fv/Fm = (Fm-Fo)/Fm; nonphotochemical quenching coefficient Y(NPQ) = 1-Y(II)-1/[NPQ + 1 + qL-(Fm/F0-1); nonphotochemical quenching parameter qN = (Fm-Fm′)/(Fm-F0); PS(II) effective electron yield Y(II) = (Fm'—F)/Fm'; and photochemical bursting coefficient qP = (Fm'—F)/(Fm'—Fo').

### Determination of antioxidant enzyme activities, reactive oxygen species and malondialdehyde contents

The NBT method was used for the determination of SOD activity (Agami and Mohamed 2013). POD activity was determined using guaiacol as a substrate (Chen and Zhang 2016). CAT activity was determined using the hydrogen peroxide method (Du et al. 2017). The H_2_O_2_ content was determined using the iodometric method (Wei et al. 2009). In situ H_2_O_2_ staining of the roots and leaves was performed using the 3,3'-diaminobenzidine (DAB, 1 mg/mL, pH = 3.8) method as described by Xia et al. (2009). In situ O_2_^.−^ staining of the roots and leaves was performed using nitroblue tetrazolium (NBT, 0.5 mg/ml in 50 mM PBS, pH = 7.8) as described by Ahammed et al. (2013). The ROS fluorescent probe DCFH-DA (Beyotime, China) was used to detect endogenous ROS levels in the root tips according to the manufacturer's instructions (excitation wavelength of 488 nm and emission wavelength of 530 nm). MDA levels were determined using the thiobarbituric acid (TBA) method (Zeng et al. 2020).

### Transcriptome analysis and RT‒qPCR analysis

Transcriptome sequencing libraries were generated using the Hieff NGS Ultima Dual-mode mRNA Library Prep Kit for Illumina (Yeasen Biotechnology (Shanghai) Co., Ltd.). After passing quality control, the libraries were analyzed on the Lumina NovaSeq 6000 platform for PE150 mode sequencing. The raw reads were further processed and analyzed using the bioinformatics platform BMKCloud (www.biocloud.net). The reference genome sequence is *Solanum lycopersicum* SL4.0_and_ITAG4.0.genome.fa. The raw data were submitted to the National Center for Biotechnology Information (NCBI) Short Read Archive (SRA) under accession number PRJNA952335. A FDR < 0.01 and log_2_ (fold change) > 1 or < -1 were used as criteria to screen the DEGs. WGCNA was performed according to the methods of Wang et al. (2023). GO network analysis was performed by association analysis between the significant modules and treatment groups using the OmicShare cloud platform (https://www.omicshare.com), and GO network visual presentation was carried out using Cytoscape v3.9.1 (Q value < 0.05).

RNA reverse transcription was performed using the cDNA synthesis kit NovoScript® Plus All-in-one 1st Strand cDNA Synthesis SuperMix (gDNA Purge, Novozymes, China). RT‒qPCR analyses were performed for three biological and technical replicates. The specific primers used are shown in Supplementary Table 7.

### Determination of phytohormones

The contents of IAA, GA, and ABA and the cytokinins *t*ZR and iP were determined using high-performance liquid chromatography (HPLC) as described by Gao et al. (2022). Briefly, approximately 1 g of tomato root was thoroughly ground with liquid nitrogen, and the powders were then immersed in 20 ml of 80% methanol (chromatographically pure) for 16 h at 4 ℃. After centrifugation at 1000 rpm for 10 min at 4 °C, the supernatant was collected. The residue was transferred to 20 ml of 80% precooled methanol and centrifuged, after which the supernatants were merged. The supernatant was evaporated at 40 °C to remove the methanol using a rotary evaporator (RE-52AA, Shanghai, China). The supernatant was extracted three times with 10 ml of petroleum ether (chromatographically pure). After adding polyvinylpyrrolidone (PVPP) to the ether phase, the mixture was ultrasonicated for 30 min and then shaken for 30 min. After centrifugation at 13,000 r/min for 10 min, the supernatant was collected. Extraction was carried out three times. Subsequently, the ester phases were evaporated by rotary evaporation at 40 °C, and 1 ml of methanol was added to dissolve the ester phases. After filtering through a 0.45 μM filter membrane, the sample was placed at 4 °C. The separation was performed on a Syncroords C18 250 × 4.6 × 5 μm liquid chromatography column (Thermo Fisher Scientific, China) with 100% methanol as mobile phase A and 0.8% glacial acetic acid as mobile phase B. A total of 10 μl of sample was injected at a flow rate of 1 ml/min, the column temperature was set at 30 ℃, the UV wavelength was 254 nm, and the sample was detected online by a Dionex UltiMate 300 Diode Array Detector (Thermo Fisher Scientific, China).

### Trace element determination

The samples were dried in an oven at 65 °C until a constant weight was reached. Subsequently, the samples were ground to powder in a mortar and immersed in nitric acid. The contents of Ni, Fe, Mn, Cu and Zn were determined using ICP‒AES (inductively coupled plasma atomic emission spectroscopy; iCAP6300; Thermo Fisher Scientific, Waltham, MA, USA).

### Statistical analysis

All the experiments were performed in triplicate, and the data were analyzed and are presented visually using IBM SPSS Statistics 26 and GraphPad Prism 9.5.1. Significant differences were determined by ANOVA and *t* tests (*P* < 0.05).

### Supplementary Information

**Additional file 1: Supplementary Fig. 1.** Transcriptome analysis. **Supplementary Fig. 2.** The soft threshold with scale independence (*left*) and mean connectivity (*right*). **Supplementary Fig. 3.** Transcriptome module maps obtained from weighted coexpression network analysis. **Supplementary Fig. 4.** Integrated network of GO catalogs in the WGCNA modules. **Supplementary Fig. 5.** Heatmap of DEGs associated with the uptake and accumulation of micronutrients in tomato roots.

**Additional file 2: Supplementary Table 1.** DEGs identified in the roots of Ni-treated tomato seedling. **Supplementary Table 2.** DEGs involved in metal ion transport. **Supplementary Table 3.** DEGs involved in phytohormone biosynthesis. **Supplementary Table 4.** DEGs involved in phytohormone signaling pathways. **Supplementary Table 5.** DEGs involved in antioxidative enzyme biosynthesis. **Supplementary Table 6.** DEGs involved in carbon/nitrogen metabolism. **Supplementary Table 7.** List of primers for RT-qPCR.

## Data Availability

Data and materials will be made available on request.

## Abbreviations

Ni: Nickel
Fe: Iron
Mn: Manganese
Cu: Copper
Zn: Zinc
GA: Gibberellic acid
ABA: Abscisic acid
IAA: Indole-3-acetic acid
JA: Jasmonic acid
SA: Salicylic acid
*t*ZR: *Trans*-Zeatin riboside
ip: Isopentenyl-adenine
HPLC: High-performance liquid chromatography
PVPP: Polyvinylpyrrolidone
NA: Nicotinamide
His: Histidine
PRX: Peroxidase
ROS: Reactive oxygen species
TR: Thiol-reductase
NO: Nitric oxide
H_2_O_2_: Hydrogen peroxide
NBT: Nitroblue tetrazolium
DAB: 3,3'-Diaminobenzidine
SOD: Superoxide dismutase
POD: Peroxidase
CAT: Catalase
TCA: Trichloroacetic acid
DCFH-DA: 2,7-Dichlorofluorescein diacetate
TBA: Thiobarbituric acid
MDA: Malondialdehyde
PR: Primary root
FW: Fresh weight
DW: Dry weight
LRs: Lateral roots
NCBI: National Center for Biotechnology Information
SRA: Short Read Archive
FDR: False discovery rate
DEG: Differentially expressed gene
WGCNA: Weighted gene coexpression network analysis
FPKM: Fragments per kilobase million
GO: Gene ontology
