# Supplementary material for: Physiological and molecular bases of the nickel toxicity responses in tomato
Source: Stress Biol. 2024 May 9;4(1):25. doi: 10.1007/s44154-024-00162-0 (PMC11082119; doi:10.1007/s44154-024-00162-0)
Supplement: Supplementary file 1 — Additional file 1: Supplementary Fig. 1. Transcriptome analysis. Supplementary Fig. 2. The soft threshold with scale independence (left) and mean connectivity (right). Supplementary Fig. 3. Transcriptome module maps obtained from weighted coexpression network analysis. Supplementary Fig. 4. Integrated network of GO catalogs in the WGCNA modules. Supplementary Fig. 5. Heatmap of DEGs associated with the uptake and accumulation of micronutrients in tomato roots. [file 44154_2024_162_MOESM1_ESM.docx]

**Supplemental figures**

**Running title:** Nickel toxicity responses in tomatoes

**Title:** Excess nickel reprograms primary metabolism and phytohormone signaling pathways in tomato seedlings

**Authors:** Hao Yu^1,2^, Weimin Li^1,2^, Xiaoxiao Liu^1,2^, Qianqian Song^1,2^, Junjun Li^1,2^, Jin Xu^1,2,*^

**Footnotes**

* Correspondence: xujin@sxau.edu.cn (J. Xu).

^1^ College of Horticulture, Shanxi Agricultural University, Taigu 030801, China

Full list of author information is available at the end of the article

**
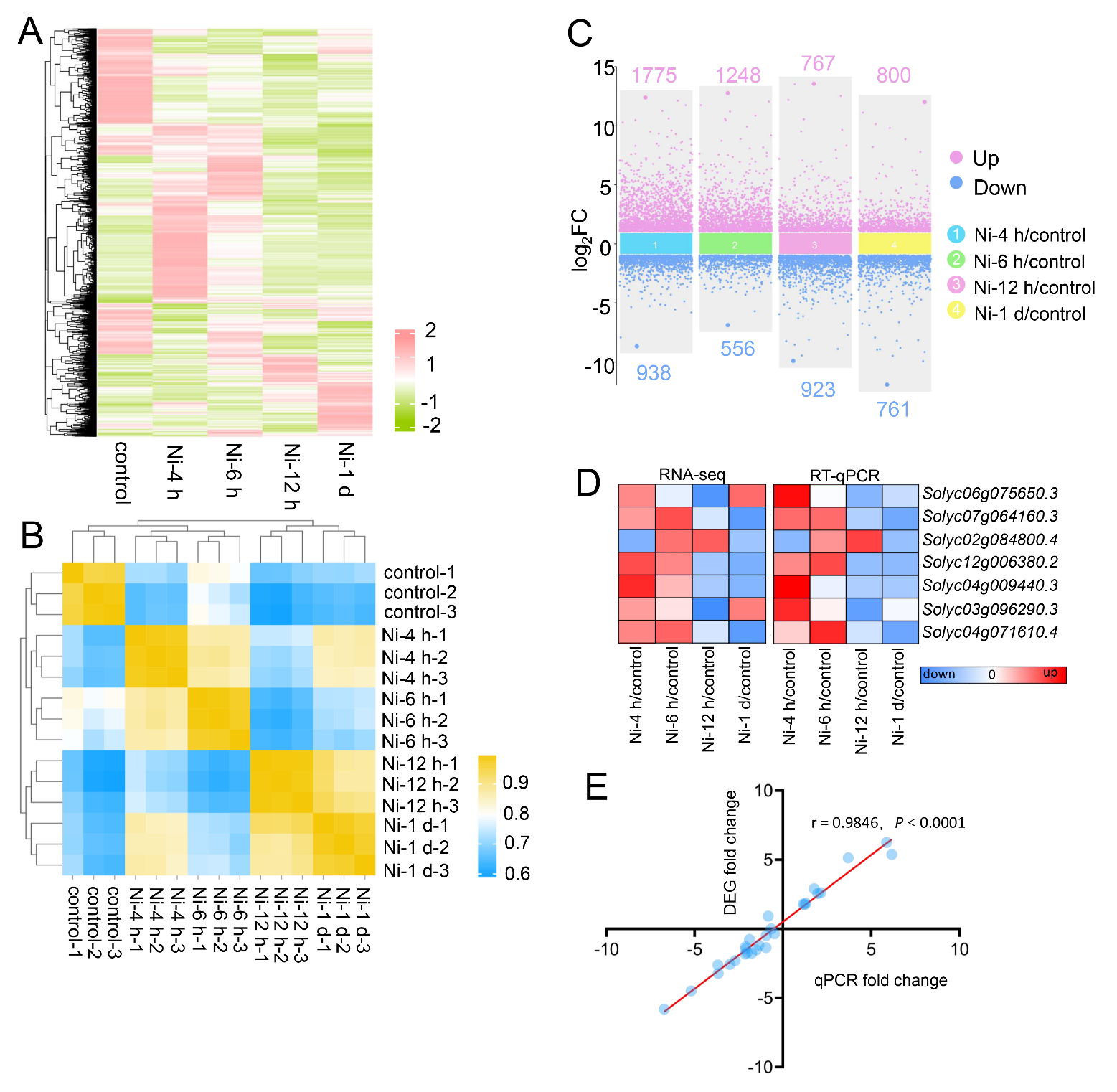
**

**Supplementary Fig. 1. Transcriptome analysis. A**, Clustering heatmap of DEGs. **B**, Intragroup correlation analysis of the transcriptome data. **C**, Multigroup difference scatter plot showing upregulated and downregulated genes. **D**, Validation of the RNA-seq data using RT‒qPCR. **E,** Reliability analysis of the RNA-seq data based on comparison with the RT‒qPCR data.


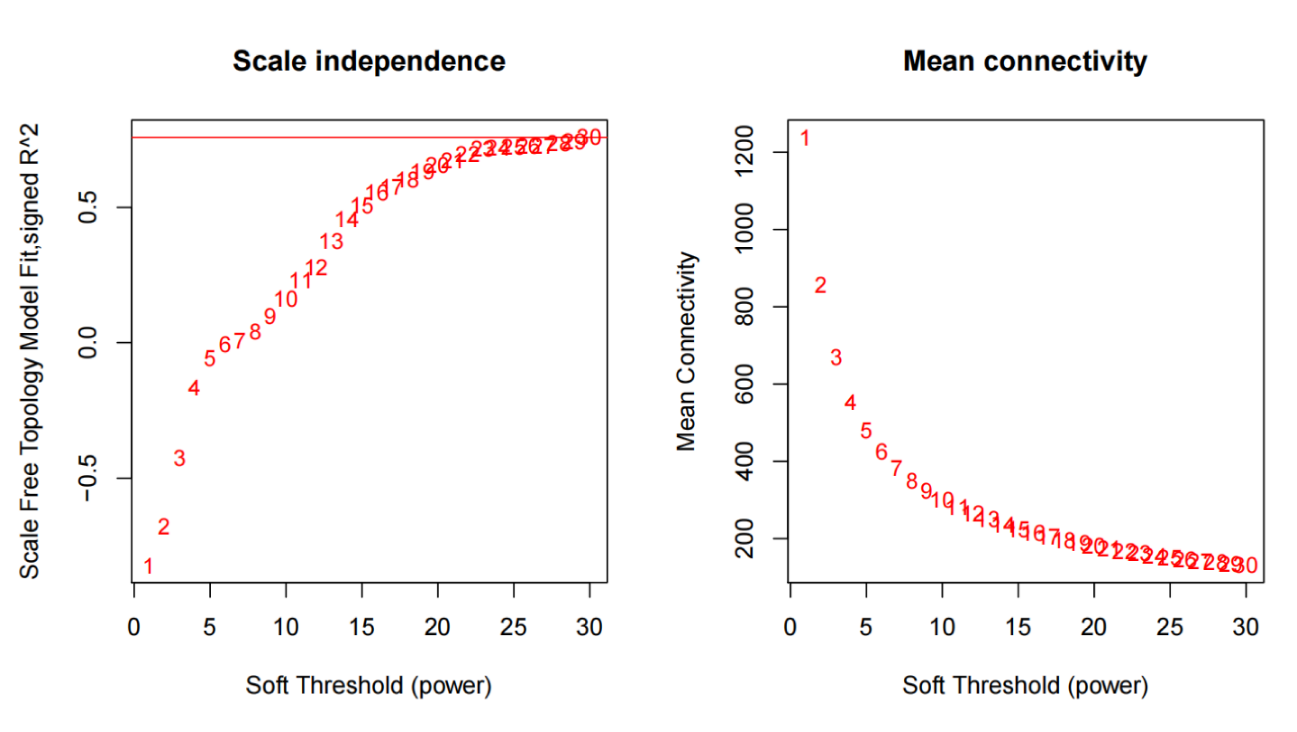


**Supplementary Fig. 2. The soft threshold with scale independence (*left*) and mean connectivity (*right*).** The default value of parameter *β* ranges from 1 to 30. The horizontal axis is the weight parameter *β*. The *β* value corresponding to the red line is the most appropriate *β* value in the experiment.


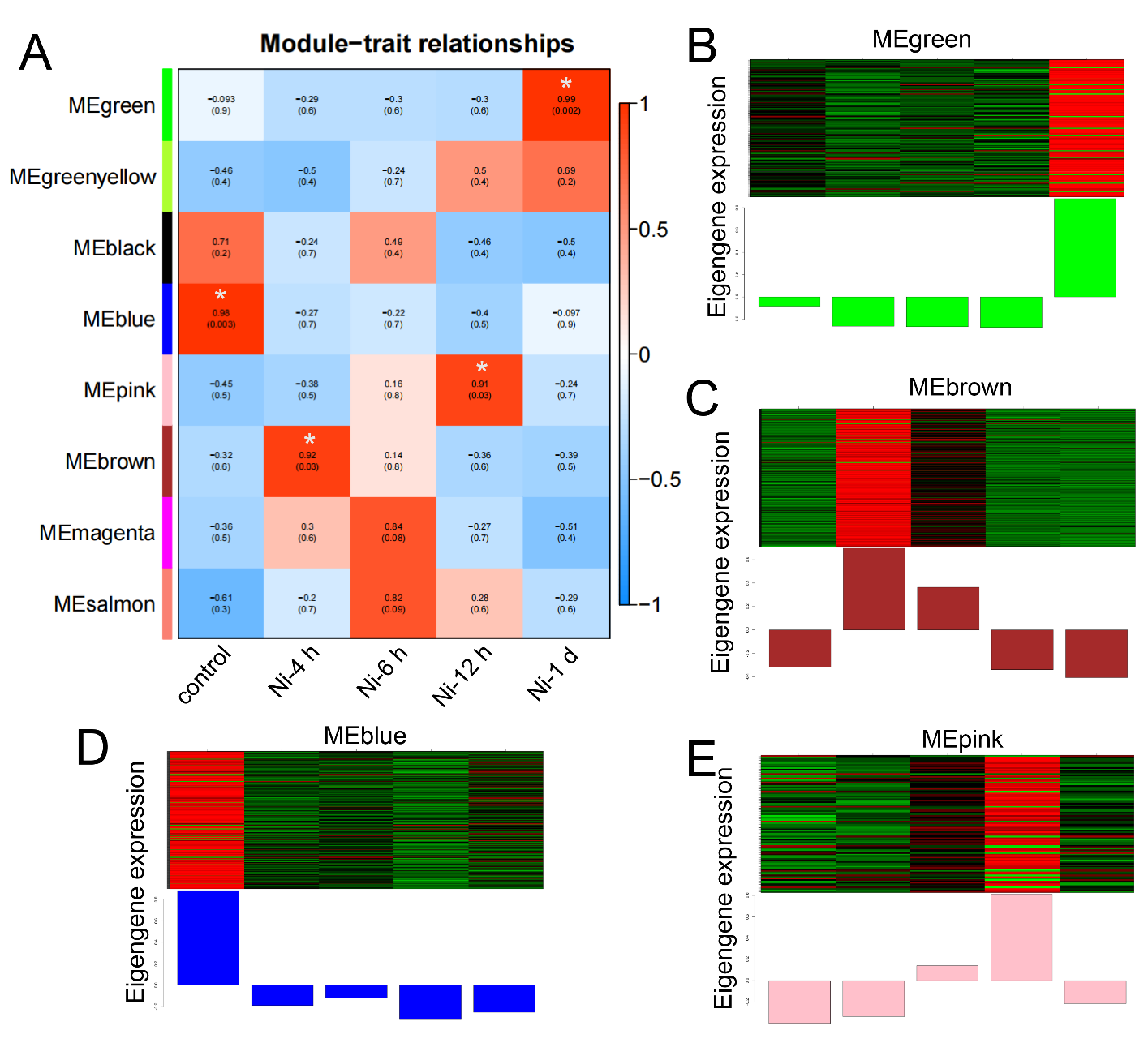


**Supplementary Fig. 3.** **Transcriptome module maps obtained from weighted coexpression network analysis.** (**A**) Correlation analysis between nickel treatments and modules at different time points. Module names are displayed on the Y-axis, and different treatments are displayed on the X-axis. The depth of the color indicates the degree of correlation (red, positive correlation; blue, negative correlation). **B-E** Heatmaps showing the expression profiles of all coexpressed genes in the (**B**) MEgreen, (**C**) MEbrown, (**D**) MEblue and (**E**) MEpink modules. The color scale indicates the expression level of the genes. The bars show consistent expression patterns of the genes coexpressed with the modules.


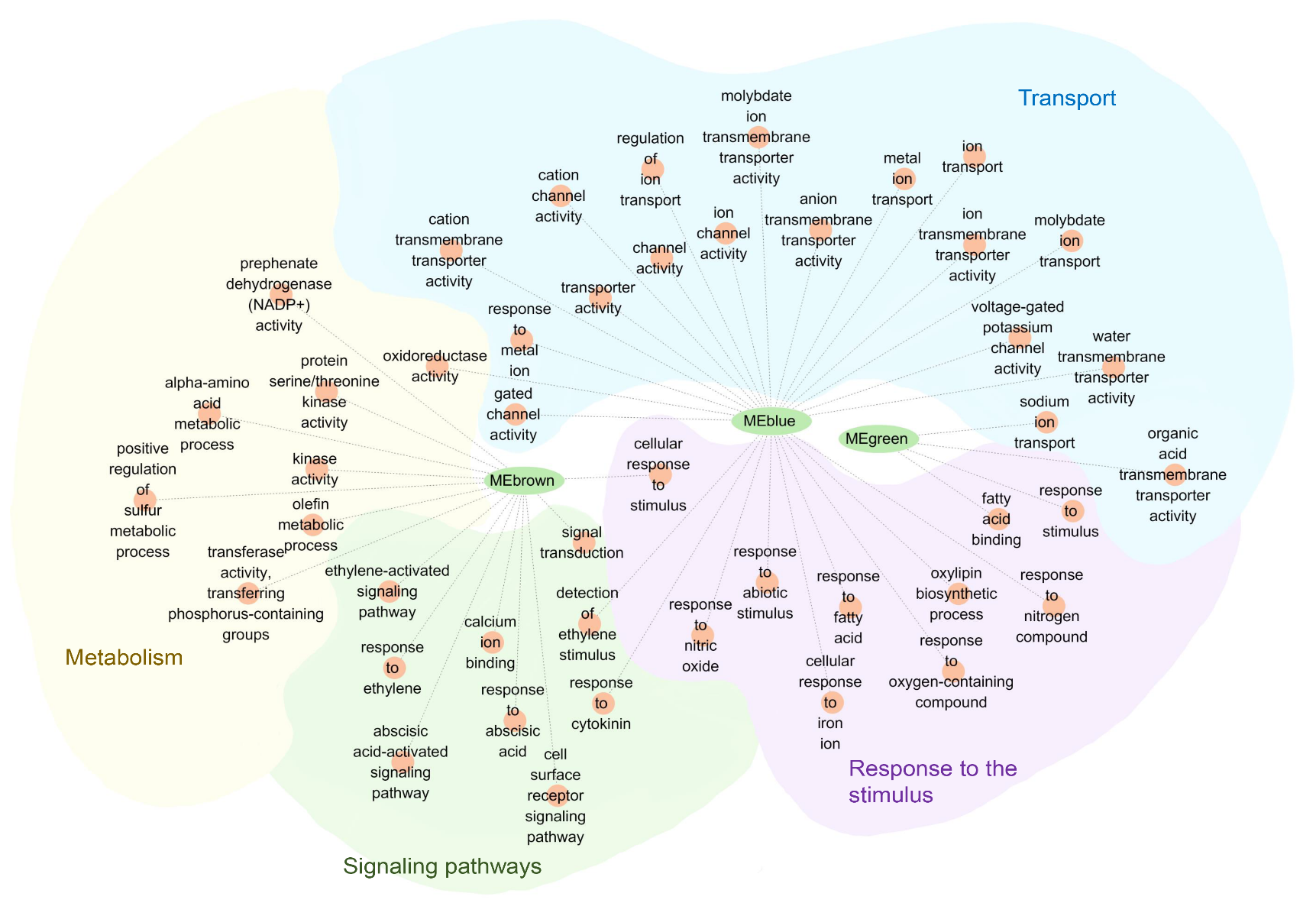


**Supplementary Fig. 4. Integrated network of GO catalogs in the WGCNA modules.** The green ovals represent the modules (MEs). Pink circles connected to the ME with dashed lines represent nodes enriched for GO terms in each module (FDR < 0.05).

**
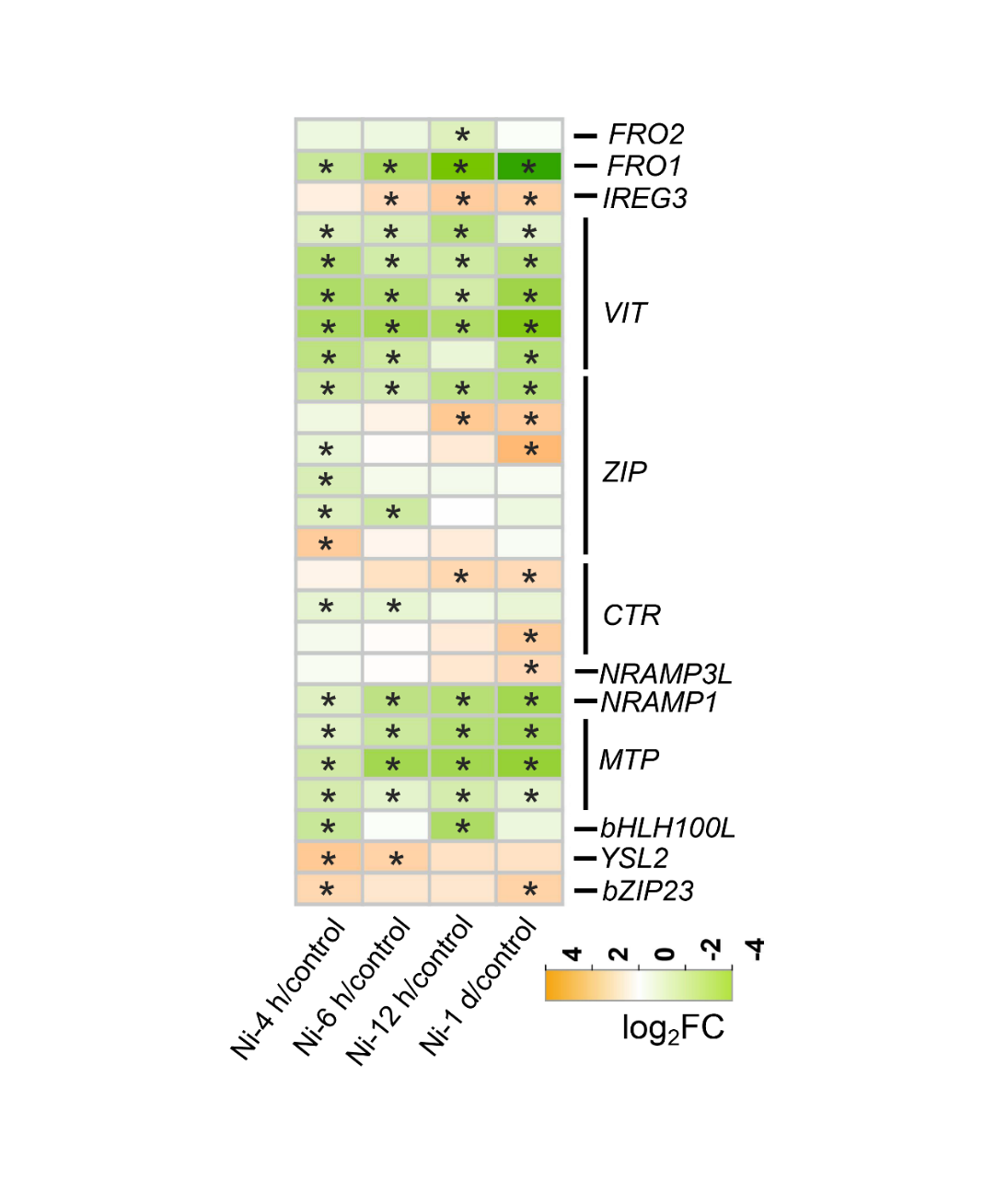
**

**Supplementary Fig. 5. Heatmap of DEGs associated with the uptake and accumulation of micronutrients in tomato roots.** The heatmaps show the gene expression patterns according to the log_2_(fold change), and the asterisks in the heatmaps represent the DEGs.
